# Cannabinoids and Inflammations of the Gut-Lung-Skin Barrier

**DOI:** 10.3390/jpm11060494

**Published:** 2021-05-31

**Authors:** Cristian Scheau, Constantin Caruntu, Ioana Anca Badarau, Andreea-Elena Scheau, Anca Oana Docea, Daniela Calina, Ana Caruntu

**Affiliations:** 1 Department of Physiology, “Carol Davila” University of Medicine and Pharmacy, 050474 Bucharest, Romania; cristian.scheau@umfcd.ro (C.S.); costin.caruntu@gmail.com (C.C.); ancab52@yahoo.com (I.A.B.); 2 Department of Dermatology, “Prof. N. Paulescu” National Institute of Diabetes, Nutrition and Metabolic Diseases, 011233 Bucharest, Romania; 3 Department of Radiology and Medical Imaging, Fundeni Clinical Institute, 022328 Bucharest, Romania; andreea.ghergus@gmail.com; 4 Department of Toxicology, University of Medicine and Pharmacy of Craiova, 200349 Craiova, Romania; 5 Department of Clinical Pharmacy, University of Medicine and Pharmacy of Craiova, 200349 Craiova, Romania; 6 Department of Oral and Maxillofacial Surgery, “Carol Davila” Central Military Emergency Hospital, 010825 Bucharest, Romania; ana.caruntu@gmail.com; 7 Department of Oral and Maxillofacial Surgery, Faculty of Dental Medicine, “Titu Maiorescu” University, 031593 Bucharest, Romania

**Keywords:** cannabinoids, inflammation, gut-lung-skin barrier, signaling pathways, inflammatory biomarkers

## Abstract

Recent studies have identified great similarities and interferences between the epithelial layers of the digestive tract, the airways and the cutaneous layer. The relationship between these structures seems to implicate signaling pathways, cellular components and metabolic features, and has led to the definition of a gut-lung-skin barrier. Inflammation seems to involve common features in these tissues; therefore, analyzing the similarities and differences in the modulation of its biomarkers can yield significant data promoting a better understanding of the particularities of specific signaling pathways and cellular effects. Cannabinoids are well known for a wide array of beneficial effects, including anti-inflammatory properties. This paper aims to explore the effects of natural and synthetic cannabinoids, including the components of the endocannabinoid system, in relation to the inflammation of the gut-lung-skin barrier epithelia. Recent advancements in the use of cannabinoids as anti-inflammatory substances in various disorders of the gut, lungs and skin are detailed. Some studies have reported mixed or controversial results, and these have also been addressed in our paper.

## 1. Introduction

The epithelial lining is the first line of defense against the abundance of aggressive factors in the environment. The integer epithelium represents a mechanical barrier, while the physiological processes occurring in and between the cells contribute to dynamic and complex protection from physical, chemical and biological agents. The immune role of the epithelial layer is supported by various cells and cytokines, which show common features between the gastrointestinal tract and the pulmonary and cutaneous systems [1,2,3]. These similarities in role and functioning have led recent research in the direction of exploring the gut-lung-skin barrier as an entity that not only shows common elements but also interactions [4]. The cross-talk between these three regions refers to various signaling molecules which are mainly involved in local and systemic inflammation [5].

This paper focuses on the roles of cannabinoids in inflammation affecting the gut-lung-skin barrier and brings forward the similarities and distinctions in the pathophysiology of the inflammatory conditions affecting these systems. The latest advancements in the field are discussed and supported by the available in vitro and in vivo evidence in the recent literature, as well as the human studies which have been undertaken to validate the effectiveness and safety of cannabinoids in the epithelial inflammation of the gut-lung-skin barrier.

## 2. The Gut-Lung-Skin Barrier

The gut-lung-skin barrier is a virtual structure encompassing the epithelium of the digestive tract, the pulmonary system and the cutaneous layer. More and more often, studies cite similarities and interactions in the immune response regulated in these structures [4,5,6]. The recently described T helper 17 (Th17) subset of cells and their regulatory pathway provide further evidence validating the unity of the immune response and regulation in the gut-lung-skin barrier [7]. These cells are capable of producing various cytokines such as granulocyte colony-stimulating factor (G-CSF) and interleukins (ILs) -8 and -22, and trigger the synthesis of effectors producing IL-17 and interferon-gamma (IFN-γ) [8]. Furthermore, Th17 cells are involved in the interactions between the microbiota of the specific tissue and the host, regulating the immune response [8]. The most prominent cytokines involved in the protection against microbial infection are IL-17 and IL-22, which increase the release of antimicrobial peptides, neutrophil recruitment and granulopoiesis while maintaining the integrity of the epithelial layer in boundary tissues [9]. When IL-17 fails due to insufficient expression, a large variety of cutaneous, pulmonary and gastrointestinal infections may occur, accompanied by significant inflammation [10,11,12]. Some infectious agents such as *Staphylococcus aureus* are capable of stimulating its expression [13]. IL-22 also contributes to the immune response against various infectious diseases of the gut-lung-skin barrier and exhibits pro-inflammatory roles by enhancing the effects of tumor necrosis factor-alpha (TNF-α), and by activating various other cytokines [9,14].

However, the gut-lung-skin barrier cross-talk in inflammation is not limited to the activity of Th17 cells. The epithelial cells produce thymic stromal lymphopoietin (TSLP), a cytokine similar to IL-7, which is involved in the allergic response and is a key factor in the onset of allergic inflammation in the skin, lungs and gastrointestinal tract [15]. TSLP is considered to be a biomarker for disruptions of the epithelial integrity, and was cited as a determining and aggravating factor in a variety of allergy models, including ovalbumin (OVA)-induced asthma, atopic dermatitis and allergic diarrhea [16].

Sustained inflammatory processes in the gut-lung-skin barrier develop similar complications, regardless of the initial location of the inflammation. Among the common comorbidities are metabolic syndrome, cardiovascular events and bone loss [5,17,18]. These may be explained by the increased production of TNF-α, which is an essential mediator of chronic inflammation and is usually accompanied by IL-4, IL-6 and IL-17, increasing the risk of the aforementioned complications, as well as favoring the appearance of other metabolic conditions [5,19,20].

Because inflammation is the cornerstone of many pathological conditions, including cancer, the possible connections and interferences between inflammatory processes in different organs and systems have substantial implications. Inflammatory conditions, regardless of their substrate or location, benefit from relatively specific management, which includes one or several anti-inflammatory drugs. However, due to adverse effects and interactions, there is a growing interest in medicinal-grade natural compounds that have demonstrated their effectiveness and usually act through dedicated receptors that are widely distributed in the human body. These substances have been labeled ‘phytochemicals’, and some examples include capsaicin, curcumin, resveratrol and cannabinoids [21,22,23,24]. Curcumin, resveratrol, gingerol, and ginsenoside have demonstrated antioxidant and anti-inflammatory effects by modulating the MAP and NF-κB pathways, making them excellent candidates in the treatment of atopic dermatitis [25]. Multiple in vivo and in vitro studies have outlined the chemopreventive properties of whole-fruit substances in skin carcinogenesis, where carotenoids, polyphenols, flavonoids and anthocyanins have demonstrated pro-apoptotic, antiproliferative, and ROS-reducing effects in basal and squamous cell carcinoma models [26,27,28,29]. Capsaicin’s effects on chemonociception were fundamental in cutaneous pathophysiology research, but its applications have extended from skin pain modulation to various local and system-wide pathologies, including malignancies [30,31,32]. Cannabinoids, in particular, show great promise due to the mediation of their actions by the dedicated cannabinoid receptors, which serve the activity of the endocannabinoid system but also respond to the administration of natural or synthetic cannabinoids.

## 3. Cannabinoids and Inflammation

Cannabinoids have been used for their anti-inflammatory properties for millennia; for instance, T’ang Shên-wei records effects such as “undoing rheumatism” or the “discharge of pus” in the 10th century A.D. work called ‘Chêng-lei pên-ts’ao’ [33]. An increasing number of studies explore their properties and interactions in a wide range of experimental models [34]. The chemical group of cannabinoids includes over 60 natural (phyto-) cannabinoids and over 150 artificial (synthetic) cannabinoids alongside the two well-known endocannabinoids anandamide (AEA) and 2-Arachidonoylglycerol (2-AG), and their few derivates [35,36]. Evidently, the process of testing and comparing the anti-inflammatory effects of these substances in inflammatory conditions is lengthy and complex. There are two G protein-coupled receptor (GPCRs) cannabinoid receptors activated by cannabinoids: CB1, which is mostly located in the central, peripheral and enteric nervous system, and is responsible, among others, for the psychoactive effects; and CB2, which is expressed by immune cells and some tissues, and is mostly involved in immunomodulation [37]. The two receptors exert distinct functions via specific signaling pathways, and different cannabinoids can activate them to various degrees; therefore, cannabinoids can vary in terms of effectiveness and potency [38]. While some anti-inflammatory properties were also described for CB1-specific agonists, their effects may also be carried through non-CB1-non-CB2 signaling, and their most common usage is the management of pain, in which they prove highly effective [39,40]. Conversely, CB2 receptors are mainly involved in regulating inflammation by modulating the levels of various cytokines [41]. A recent systematic review of in vivo studies regarding the anti-inflammatory effects of various cannabinoids concluded that the CB2 antagonist CBD is efficient in reducing inflammation, while CB1 agonists—such as THC—may be used for the alleviation of the associated pain [42].

Some cannabinoids are favorites in the race for the identification of the substance with the optimal anti-inflammatory actions and minimal adverse reactions. Cannabidiol (CBD) is one of the most commonly tested substances due to its lack of psychoactive effects and high potency in modulating the immune response and exerting anti-inflammatory properties in a variety of animal models [43,44,45]. Tetrahydrocannabinol is still tested, despite its psychoactive effects, due to its activity on the CB1 receptor which triggers specific signaling pathways in inflammation [46]. As for synthetic cannabinoids, there are multiple classes and subclasses defined by their chemical structure, which encompass a growing number of substances, developed with the intent of emulating the favorable effects while maximizing the receptor specificity and limiting side effects. These substances are agonists for CB1 and/or CB2, and usually bind to the cannabinoid receptors with higher affinity than natural compounds, showing great potential in developing new treatments [47].

The following sections explore the roles of synthetic, phyto-, and endocannabinoids in the inflammations of the gastrointestinal tract, the pulmonary apparatus and the skin.

## 4. Cannabinoids and Gut Inflammations

The discovery of the intestinal endocannabinoid system was encouraged by numerous reports of the beneficial effects of administering cannabinoids for various digestive tract disorders [48]. This system plays various roles in the homeostasis of the gastrointestinal tract, including the regulation of secretion, sensitivity and motility, as well as upholding the integrity of the gut epithelial barrier [48,49]. Physiological and pharmacological studies have investigated the interactions between endocannabinoids and various receptors, and have concluded that these substances may play a role in modulating intestinal inflammation and cellular proliferation [50]. These findings led to the consideration of phyto- and artificial cannabinoids as candidates for the treatment of intestinal inflammatory disorders, such as irritable bowel syndrome (IBS), inflammatory bowel disease (IBD) and Crohn’s disease [51].

In the gastrointestinal tract, CB1 and CB2 receptors are located in the myenteric and submucosal neurons (mostly CB1) as well as the inflammatory and epithelial cells (mostly CB2) [52]. Besides GPCRs, cannabinoids may also bind to the transient receptor potential cation channel subfamily V member 1 (TRPV1) receptors in the capsaicin-sensitive sensory nerves located in the digestive tract wall, especially in the mucosa, muscle layers and blood vessels, but also in epithelial cells [53]. Furthermore, cannabinoids may bind to G protein-coupled receptor (GPR) type 55, which was identified in smooth muscle cells and peroxisome proliferator-activated receptor alpha (PPAR-α), located in blood vessels and smooth muscle cells, and also to GPR119, which is expressed in the digestive mucosa and enteroendocrine cells [54,55,56].

### 4.1. In Vitro Studies

An in vitro study on Caco-2 cells demonstrated that adding AEA or cannabidiol (CBD) alongside IL-17A when incubating the cell monolayers for 48 h prevents mucosal damage and the changes in the epithelial permeability associated with inflammation [57]. In a similar study using the same cell types, delta-9-tetrahydrocannabinol (THC) and CBD were also able to reduce the intestinal permeability induced by IFN-γ and TNF-α, while AEA and 2-AG enhanced the cytokine-induced permeability [58]. This leads to the conclusion that the effects are CB1-related, and that the specific agonistic effects of various cannabinoids trigger specific effects [58,59]. These findings were validated ex vivo, in inflammatory and hypoxic states, on Caco-2 cells harvested from colorectal resections [60].

Improving on this experimental model, Couch et al. tested the effects of CBD and palmitoylethanolamide (PEA) on colon explants from patients with inflammatory conditions; these cannabinoids elicit anti-inflammatory properties by preventing the increased cytokine production and reducing the intracellular signaling phosphoprotein levels [61]. The anti-inflammatory properties of phytocannabinoids have been investigated using fresh and baked *C. sativa* flowers, and it was revealed that tetrahydrocannabinolic acid (THCA) is the main active compound and is able to reduce IL-8 levels in HCT116 colon cancer cells pretreated with TNF-α at concentrations of 114–207 μg/mL, partially via the GPR55 receptor [62]. The same study further confirmed the anti-inflammatory effects on patients suffering from IBD, through COX-2 and matrix metalloproteinase (MMP)-9 mediated mechanisms, and showed that THCA exerts superior effects and less cytotoxicity than CBD on these cell lines [62].

Another important anti-inflammatory property is the maintenance of the epithelial barrier’s integrity. CBD is able to preserve the mucosal integrity in Caco-2 cells at concentrations of 10^−7^ to 10^−9^ M, opposing the effects of *Clostridium difficile* toxin A in an inflammation experimental model; this activity is CB1-mediated, as the use of AM251, a CB1 antagonist, inhibited the observed effects [63]. An in vitro study on HT29 cells showed that AEA, methanandamide (mAEA), and arachidonylcyclopropylamide (ACPA) stimulate CB1-dependent wound closure even in low nanomolar concentrations [64]. These findings consolidate the hypothesis of the intrinsic protective effect of endocannabinoids against intestinal barrier disruptions.

Conversely, CB2 receptors have also been implicated in mediating the anti-inflammatory effects of β-Caryophyllene on oral mucositis, which is characterized by the decrease of TNF-α, IL-1β, IL-6 and IL-17A [65]. Furthermore, Matalon et al. showed, in their recent paper, that the synthetic CB2 agonist JWH-133 is able to reduce MMP-9 and IL-8 levels in inflamed colon biopsies from patients with IBD. These findings draw further attention to the specific involvement of the two cannabinoid receptors in the various facets of inflammation. There is extensive evidence that CB1 is normally expressed in the intestinal epithelium, while CB2 expression seems to be stimulated by inflammatory conditions, supporting its attributed role in the regulation of inflammation, cell growth and the immune response [64,66,67,68].

### 4.2. In Vivo Animal Studies

The effectiveness of cannabinoids in inflammatory diseases has been demonstrated on various animal models. Couch et al. published a comprehensive meta-analysis presenting undeniable evidence that cannabinoids prove to be effective in intestinal inflammatory conditions [69]. The disease activity index (DAI) score and levels of myeloperoxidase (MPO) activity were the criteria of choice for the measurement of the effects of various synthetic and phyto-cannabinoids.

In an in vivo murine model of IBD induced by the intracolonic administration of dinitrobenzene sulphonic acid (DNBS), cannabigerol (CBG) reduced the expression of inducible nitric oxide synthase (iNOS), as well as the levels of IL-1β, IL-10 and IFN-γ, and the activity of myeloperoxidase, while increasing the activity of superoxide dismutase [70]. CBD appears to elicited analogous effects in a parallel study [71]. Using the same experimental model, Pagano et al. noted similar effects when using a combination of fish oil, CBD and CBG [72]. The association of fish oil and CBD enhances their anti-inflammatory effects, which occur at lower doses (20 mg and 0.3–10 mg/kg, respectively) than the per se administration of the two substances; these findings were reported in mice with induced colitis, in which the combination of fish oil and CBD decreased the myeloperoxidase (MPO) activity, DAI score, intestinal permeability, and levels of IL-1β and IL-6 [73].

An additional key factor in the evolution and management of intestinal inflammations is the gut microbiome. The interactions between various microbes and the intestinal mucosa seem to play an important role in the local immune response to various conditions including IBD and Crohn’s disease [74]. CBD is able to induce changes in the gut microbiota, apparently independently from its effects on local inflammation [73]. This is of interest because there is evidence that the endocannabinoid system connects the intestinal microbiome with the physiological processes occurring in the adipose tissue, through regulatory pathways [75]. Furthermore, and more importantly, it appears that the alteration of the balance between the endocannabinoid system and the microbiota can negatively impact the integrity of the intestinal barrier [76].

In vivo studies on animal models confirmed that cannabinoids protect the intestinal barrier’s integrity. In this regard, the role of CB1 has been validated using a knockout mice model which showed that CB1 is responsible for the intestinal physiological response to inflammation, leading to the secretion of IgA and the regulation of intestinal permeability, among other effects [77]. Synthetic cannabinoids can further enhance these properties. Cao et al. provoked acute lesions of the gastrointestinal mucosa in rats by inducing acute pancreatitis and then showed that synthetic cannabinoid HU210, a non-selective CB agonist, reversed the morphological and serum anomalies, demonstrating its anti-inflammatory properties [78]. A later study showed that HU210 protects the intestinal mucosa in a murine model of ulcerative colitis, most likely through the toll-like receptor 4 (TLR4) and mitogen-activated protein (MAP) signaling pathways [79]. Another synthetic cannabinoid, abnormal cannabidiol, is an isomer of cannabidiol that exerts its effects via non-CB1-non-CB2 signaling and elicits protective effects against chemically induced colitis in mice, stimulating wound healing while inhibiting neutrophil recruitment [80].

### 4.3. Human Clinical Trials

Up to the moment of this review, limited information is available regarding the in vivo effects of cannabinoids on intestinal inflammatory conditions in humans. The outcome predictors in IBD trials usually include DAI, C-reactive protein (CRP), rapid fecal calprotectin (FC), the Mayo score and the Crohn’s Disease Activity Index (CDAI), and the correct interpretation of the results relies on quality preparation for colonoscopy [81,82,83].

A recent paper by Kienzl et al. gathered retrospective data from patients suffering from IBD that used cannabis to alleviate symptoms [76]. Most studies included in the analysis showed that cannabis users benefited from symptom relief, improved DAI, and better quality of life. However, despite the overall good tolerance, the adverse reactions of cannabis inhalation are to be considered [76,84]. Furthermore, in one survey, patients with Crohn’s disease presented a higher risk for surgery [85]. The legalization of medicinal marijuana did not affect its use in patients with IBD, despite an upward trend in the use of cannabis in the tested population throughout the previous 5 years [86].

A randomized clinical trial on healthy subjects performed by Couch et al. tested the effects of PEA and CBD on the regulation of the increased intestinal permeability induced by the pro-inflammatory effects of aspirin [87]. A dose of 600 mg of PEA and CBD, respectively, managed to prevent the increase in intestinal permeability caused by 600 mg of aspirin, evidenced by the measurement of lactulose and mannitol excretion in the urine.

Two randomized clinical trials were published by Naftali et al., in 2013 and 2017, which tested the effects of inhaled THC and oral CBD, respectively, in Crohn’s disease [88,89]. While THC showed some clinical benefits, CBD failed to improve the DAI in active Crohn’s disease. However, both trials received some criticism for methodological flaws, small patient groups and various other biases [69,90].

Two other randomized placebo-controlled studies studied the effects of cannabinoids in ulcerative colitis. Irving et al. tested a combination of THC and CBD capsules that were administered in increasing doses, and found that while the remission rates were similar to the placebo, the cannabinoid capsules offered subjective improvements to the patients’ quality of life, despite mild to moderate side effects [91]. Naftali et al. tested the effects of inhaled THC and recorded significant improvements in the DAI, as well as decreases in the CRP and FC while registering no serious adverse effects [92].

These findings are encouraging, and the favorable in vitro results and animal studies observations warrant that further human clinical trials despite the low number of studies published to date. Furthermore, synthetic cannabinoids may demonstrate superior effectiveness and similar or fewer side effects, and could represent a major improvement in the treatment of intestinal inflammatory diseases.

## 5. Cannabinoids and Lung Inflammatory Conditions

Cannabis has been known for its recreational use for millennia, so it is not surprising that the effects of cannabinoid inhalation have been thoroughly documented in many studies. Though it is difficult to distinguish from concurrent chronic exposure to tobacco smoke, which is common, cannabis smoke inhalation causes some specific effects in the pulmonary system; it decreases the antimicrobial activity and cytokine production, and increases sputum production, coughing and possibly the risk of lung cancer [93,94]. Furthermore, marijuana smoke induces epithelial hyperplasia, cellular disorganization, cell atypia and fibrosis [95,96]. In vitro studies seem to confirm these findings at a cellular level, as cannabinoids have demonstrated immunosuppressive properties and profibrotic effects [97,98]. However, in vivo studies using the direct administration of pharmaceutical-grade cannabinoids have revealed benefic effects on several inflammatory conditions, inducing a decrease in the recruitment of inflammatory cells, the suppression of cytokines, and an overall improvement in mortality [99]. These anti-inflammatory and immunomodulatory properties have prompted researchers to investigate the potential of cannabinoids in the management of coronavirus disease (COVID-19) infection [100,101,102]. However intriguing, the risks of drug interactions and partial inhibition of the immune response seem to outweigh the potential untested benefits in this condition, and the use of these substances is not recommended in this case, according to some authors [103]. Therefore, the main application and use of cannabinoids appear to be inflammatory diseases, and there is a great interest in this topic, which has encouraged numerous studies in the field.

No cannabinoid receptors have been isolated in vivo in the epithelial cells of the lungs; however, CB1 receptors have been identified in the nerve endings of the airways [104]. Both CB1 and CB2 receptors are expressed by eosinophils, monocytes and monocyte-derived macrophages [99,105]. CB1, CB2 and TRPV1 have been identified in situ and in vitro at the protein level in airway epithelial cells; however, the impact of these findings on the biology of respiratory inflammations remains unclear [106].

### 5.1. In Vitro Studies

A study on A549 cells has shown that marijuana smoke causes mitochondrial damage, impairing the energetic metabolism of the cell [107]. Furthermore, using the ECV304 cell line, Sarafian et al. demonstrated that even short exposures to marijuana smoke cause an increase in reactive oxygen species (ROS) production capable of inducing necrotic cell death, and the effects seem to be mainly attributed to the gaseous phase—not the particulate phase—of the smoke [108].

However, studies focused on the direct effects of the substances isolated from plants showed controversial results. In a study on multiple human cell lines, including eosinophils and natural killer (NK) cells, both THC and CBD decreased the levels of IL-8, macrophage inflammatory protein (MIP)-1, IFN-γ and TNF-α, thus demonstrating anti-inflammatory properties [109].

The anti-inflammatory effects of cannabinoids were cited in the context of septic lung injury. The highly CB2-selective synthetic cannabinoid HU308 decreased the levels of TNF-α, IL-18, IL-1β, and NLR family pyrin domain containing 3 (NLRP3) in RAW264.7 macrophages in lipopolysaccharide (LPS)-induced inflammation, effects which were also observed in an in vivo animal model of acute lung injury (ALI) in the same study [110].

Additional studies revealed a more complex interaction between cannabinoids and the immune system. In a study on macrophages, lung fibroblasts and epithelial cells, Muthumalage et al. showed that CBD reduced the levels of IL-8, monocyte chemoattractant protein (MCP)-1 and nuclear factor-kappa B (NF-κB) activity when they were increased by an LPS-induced inflammatory state [111]. However, when co-administered with dexamethasone, CBD demonstrates antagonistic effects, possibly due to receptor competitivity and pharmacological interactions in the signaling pathways [111,112].

Cannabinoids have also demonstrated anti-angiogenic properties, with possible applications in inflammation and cancer. The synthetic cannabinoids arachidonyl-2′-chloroethylamide (ACEA) and JWH-133 are selective CB1 and, respectively, CB2 agonists which inhibit the LPS-induced production and release of vascular endothelial growth factors A and C, angiopoietins 1 and 2, and IL-6 from human lung macrophages [113].

### 5.2. In Vivo Animal Studies

The observational studies citing the toxic effects of marijuana smoke on the pulmonary system have prompted in vivo studies on animals to further investigate the extent of the effects. As expected, animal studies have evidenced a large variety of lung lesions, including neutrophil, lymphocyte, and macrophage infiltration, goblet cell hyperplasia, endothelial proliferation, emphysema and airway hyperresponsiveness (AHR) doubled by increased cytokines and inflammatory pathway activation [114]. Interestingly, when the cannabinoids were removed from the inhaled smoke, the alveolar inflammation and wall thickening, pneumonitis, tracheobronchial fibrosis, inflammation and sputum excess were still observed [115]. These findings prompted the need for research using *per se* cannabinoids in controlled studies.

A study investigating the effects of the synthetic cannabinoid CP55,940, a full CB1 and CB2 agonist, in C57BL6/J mice showed that the oropharyngeal instillation of the compound caused CB1 activation and the subsequent increase of TNF-α, IL-1β, IL-6, C-C Motif Chemokine Ligand 2 (CCL) 2 and 3, C-X-C motif chemokine ligand 10 (CXCL10), and various pro-inflammatory transcription factors [116].

However, the in vivo effects of cannabinoids were most commonly investigated in animal-induced inflammatory conditions, most commonly using LPS to induce ALI. The intraperitoneal administration of CBD is able to reduce TNF-α, IL-6, MCP-1 and MIP-2 in the bronchoalveolar lavage fluid (BALF), as well as being able to decrease the lung myeloperoxidase activity and the pulmonary infiltration of leukocytes [117,118]. Other cannabinoids exhibited similar effects. WIN 55,212-2, THC and AEA caused a dose-dependent decrease of TNF-α and neutrophil recruitment in BALF after intranasal administration, while PEA only decreased the TNF-α levels [119]. Conversely, pro-inflammatory effects were cited for CBD in this animal model of ALI. The oral administration of CBD amplified the production of TNF-α, IL-5, IL-23 and G-CSF in C57BL/6 mice, while also increasing the infiltration of neutrophils and monocytes in the BALF [120].

Another animal model of lung inflammation was used by Arruza et al. to test the effects of CBD on newborn piglets with hypoxic–ischemic brain damage [121]. CBD decreased IL-1, the protein content and leukocyte infiltration in the BALF and the extravascular lung compartment; interestingly, the authors identified that the serotonin 1A (5-HT_1A_) receptor participated in the mediation of these effects.

Some pulmonary anti-inflammatory effects of cannabinoids appear to be carried out through non-cannabinoid receptors. Tauber et al. showed that WIN55,212-2 causes a decrease in MMP-9 production in mice with lung inflammation induced by cigarette smoke via the extracellular signal-regulated kinase (ERK) signaling pathway consecutive to TRPV1 activation [122].

Asthma is a serious pulmonary disease with an important associated inflammatory component. Mice with ovalbumin (OVA)-induced asthma were treated with 5 mg/kg intraperitoneal CBD, and a reduction of TNF-α and ILs 4, 5, 6 and 13 was observed [123]. In the same animal model, CBD also decreased AHR and the collagen fiber content alongside a decrease of the inflammatory markers in the BALF [124]. Conversely, JWH-133 caused an increase in eosinophil migration, chemotaxis and ROS generation, aggravating the AHR [125]. Furthermore, a study using a CB2 knockout mouse model showed that CB2 activation triggers pro-inflammatory effects with increased IFN-γ production by pulmonary NK cells in the BALF [126].

Cannabinoids are also able to modulate the inflammation related to various infectious diseases. In a series of articles, Tahamtan et al. showed that the activation of CB1 and CB2 receptors by JZL184 and JWH-133, respectively, decreased the production of cytokines and the influx of cells, alleviating lung pathology in Balb/c mice infected with the respiratory syncytial virus (RSV) [127,128]. THC also exhibits anti-inflammatory properties in mice with influenza infection via CB1 and/or CB2 activation by decreasing the levels of IL-17 and IFN-γ, as well as the macrophage infiltration in the BALF; however, THC also caused immunosuppression by decreasing the recruitment of CD4+ and CD8+ T-cells and macrophages with a subsequent increase of the viral load [129,130]. THC is capable of reducing the cell proliferation and levels of IFN-γ, inhibiting the phosphatidylinositol 3-kinase/protein kinase B (PI3K/Akt) pathway and decreasing the *Staphylococcal* enterotoxin B-induced lung toxicity in C3H/HeJ mice [131]. PI3K/Akt pathway activation was also achieved in C57BL/6 mice treated with JWH-133 for paraquat-induced ALI, causing a CB2-mediated decrease of TNF-α, IL-6 and MPO activity while improving lung function [132].

JWH-133 reduces the serum and tissue levels of TNF-α, IL-6 and IL-1β in a polymicrobial sepsis model in rats while increasing the IL-10 levels [133]. Additionally, neutrophil recruitment, bacteremia and lung injury are decreased when CB2 synthetic agonist GP1a is used in septic C57BL/6J wild-type mice [134]. Other CB2 agonists, such as melilotus, exhibit similar effects in this animal model, while also decreasing neutrophils and lymphocyte infiltration and blocking NF-κβ activity [135].

Various other pulmonary disease models have been used to evaluate the efficacy of cannabinoids. A dose of 1 mg/kg of JWH-133 improves neurogenic pulmonary edema at 24 h after subarachnoid hemorrhage in rats, decreasing the MPO activity and leukocyte infiltration while improving the lung permeability and tight junction protein levels [136]. Furthermore, AEA demonstrated anti-inflammatory effects by increasing the expression of heat shock proteins (HSP) 25 and 70 in the lungs of rats injected with 1 mg/kg AEA [137]. Moreover, the oral or intraperitoneal administration of β-Caryophyllene prevents neutrophil infiltration and the decreased production of IL-12, NO, leukotriene B4 and CXCL1/keratinocytes-derived chemokine (KC) in C57Bl/6 mice with *Mycobacterium* bovis-induced pulmonary inflammation [138].

### 5.3. Human Clinical Trials

The usage of cannabinoids for pulmonary disorders in humans is encumbered by numerous reports of lung injury, pneumonia and respiratory depression related to recreational use, especially of synthetic cannabinoids [139,140,141]. The respiratory failure was assumed to be CB1-mediated via the mitogen-activated protein kinase (MAPK) pathway and aggravated by cumulative central nervous system depression; however, due to small sample sizes and high bias risks more information is needed to confirm these findings [142].

Genetic studies have shown that mutations of the Q63R variant of the CB2 receptor increase the severity of acute infections with RSV in children, confirming the role of cannabinoids in modulating the immune response and carrying on their known anti-inflammatory effects [128].

One of the few trials investigating the role of cannabinoids in lung inflammations is the recently published randomized controlled trial (RCT) on the use of lenabasum in patients with cystic fibrosis [143]. This Phase 2 trial showed fewer pulmonary exacerbations, a decrease in Immunoglobulin G and IL-8 levels, and a significant reduction in neutrophil and eosinophil infiltration in the sputum of patients taking 1 or 5 mg lenabasum daily for a month. An RCT investigating the anti-inflammatory effects of smoked cannabis in the pain and inflammation of patients with radiated lung cancer is still in Phase 1 [144].

A clinical trial published in 1973 showed that THC inhalation causes bronchodilation in healthy subjects [145]. Subsequently, several studies emerged attempting to apply these beneficial effects to patients with various inflammatory lung conditions. However, the results of the ensuing studies were not substantial. A clinical trial investigating the role of inhaled cannabis in the management of advanced chronic obstructive pulmonary disease showed no benefits in terms of lung function and exercise performance [146]. Furthermore, in their study, Gong et al. showed that the oral administration of 2 mg nabilone does not produce significant bronchodilation in asthmatic patients compared to a placebo [147]. In a previous study, THC was shown to be unsuitable for clinical use in asthma, because when it was administered in aerosols it produced bronchodilation in some asthmatic patients but caused bronchoconstriction, coughing and discomfort in others [148]. Oral THC did not show better results in asthmatic patients because it caused inconsistent bronchodilation, central nervous system effects and, in some cases, bronchoconstriction [149].

Positive results were obtained in a clinical trial testing the benefits of using a vaporizer to improve respiratory symptoms in frequent cannabis smokers [150]. This suggests that finding alternate vehicles of administration may improve the clinical results in future studies.

## 6. Cannabinoids and Inflammatory Skin Disorders

Phytochemicals have been increasingly employed in skin disorders as emerging data demonstrate their utility. Cannabinoids and their receptors are regarded with increasing interest for their implications in skin pathology, especially in the field of inflammatory skin disorders. While some action mechanisms are still unclear, encouraging data is becoming available as a result of a multitude of in vivo and in vitro studies investigating this topic [151]. Based on the reported anti-inflammatory properties, the application of cannabinoids has been attempted for various conditions such as acne, psoriasis, atopic dermatitis and even cancer [152]. Among the most common tested substances, CBD is preferred due to its lack of psychoactive effects, and there is evidence that it is effective in various skin inflammations, despite the incomplete understanding of its effects and interactions in molecular signaling pathways [153].

The simple and effective topical administration of cannabinoids on skin lesions is helpful not only in ensuring substance delivery to the inflammation site but also in observing local adverse effects [154]. The additional anti-aging, anti-oxidative and antitumoral effects provide supplementary benefits in the use of cannabinoids in other diseases that are associated with inflammation, including cancer [155,156]. However, many of the studies are preclinical, and there are very few trials free of bias with a large enough number of participants to be considered high-quality [157]. Nevertheless, the in vitro and animal models support further research due to the uncovered effects of cannabinoids on inflammatory cells, cytokines and signaling pathways, which are mediated to various degrees by CB receptors, depending on the affinity and effectiveness of the tested substance [156].

The cannabinoid receptors are widely dispersed in the skin. CB1 and CB2 receptors have been identified on nerve fibers, keratinocytes and mast cells [158]. CB1 was isolated in the hair follicles, while CB2 was found in sebocytes [159,160]. The intensely studied TRP channels have been identified in the sensory nerve endings, keratinocytes, endothelial cells, mast cells and dendritic cells [161,162].

### 6.1. In Vitro Studies

One of the key elements of the skin inflammatory process is the array of actions performed by the multitude of cytokines that are involved in the response to skin barrier disruption, but also in immunity, apoptosis, and even the development and progression of skin cancer [163,164,165,166].

An in vitro experiment in a larger study performed by Karsak et al. investigating the impact of the endocannabinoid system on allergic contact dermatitis (ACD) revealed that HaCaT keratinocytes with contact hypersensitivity conditions induced via polyinosinic:polycytidylic acid express an upregulation of CB1 and a downregulation of CB2 receptors [167]. An ensuing study by Petrosino et al., using the same experimental model, showed that PEA and AEA are upregulated in these conditions, while exogenous PEA causes a decrease of MCP-2 expression [168]. Continuing their previous work on this in vitro model, Petrosino et al. showed that CBD inhibits the production of MCP-2, but also of interleukins 6 and 8 and tumor necrosis factor-alpha (TNF-α) while increasing the endogenous levels of AEA [169].

While they are not considered to have anti-inflammatory effect per se, cannabinoids have demonstrated the ability to inhibit keratinocyte cell proliferation, thus limiting the pro-inflammatory roles of these cells [170]. Wilkinson et al. showed that THC, cannabinol (CBN), CBD and CBG inhibit the proliferation of human papillomavirus (HPV)-16 E6/E7 transformed human skin keratinocytes in an in vitro model for psoriasis [171]. The synthetic CB1 agonist ACEA inhibited the upregulation of keratins K6 and K16 in isolated human skin samples of psoriasis lesions [172].

The cannabinoids’ property of decreasing pro-inflammatory cytokine concentrations was also evidenced in an in vitro study performed by Robinson et al. on mononuclear cells from the peripheral blood of dermatomyositis patients treated with ajulemic acid (AJA) [173]. In their paper, the authors cite a significantly decreased concentration of TNF-α, but also of IFN-α and -β in the cells treated with ajulemic acid compared to untreated cells. These findings further indicate that cannabinoids decrease the production of cytokines in inflammatory skin disorders.

A study on SZ95 human sebocyte cultures performed by Oláh et al. demonstrated that CBD can prevent the increase of TNF-α when sebocytes are stimulated with linoleic acid and testosterone in an in vitro acne model [174]. Furthermore, CBD also decreased the expression of IL-1β and IL-6 when the sebocytes were stimulated with lipopolysaccharides, a finding that suggests a potential positive effect of CBD in the treatment of acne vulgaris. The same study identified that the anti-inflammatory effects of CBD are mediated by the Tribbles homolog (TRIB3)-NF- κB pathway via A2a adenosine receptors, yielding important insight into the action mechanisms of cannabinoids.

The anti-inflammatory effects of cannabinoids are also extended to the inflammatory milieu of tumors, as demonstrated in an in vitro study performed by Maor et al. on Kaposi’s sarcoma-associated herpesvirus-infected primary human dermal endothelial cells in an in vitro model for Kaposi sarcoma [175]. The authors reported that CBD demonstrates anti-proliferative effects by blocking the expression of growth-regulated oncogenes (GRO-α), as well as of the viral G protein-coupled receptor and vascular endothelial growth factor C and its receptor. GRO-α is a chemokine involved in angiogenesis, tumorigenesis and inflammation, suggesting that the inflammatory milieu plays a role in Kaposi cancer development, and that CBD may also demonstrate anti-tumoral effects through its anti-inflammatory properties [176].

### 6.2. In Vivo Animal Studies

VCE-004.8, a synthetic agonist for PPAR-γ and CB2 receptors, demonstrated anti-inflammatory properties in an in vivo study on mice with bleomycin-induced scleroderma performed by del Río et al. [177]. The non-thiophilic, fully substituted CBD quinol derivative labeled VCE-004.8 decreased the expression of IL-1β and IL-13, and prevented macrophage infiltration and mast cells degranulation, thus indirectly reducing the concentration of Transforming Growth Factor-beta (TGF-β), a key factor in fibrosis development. Another study on the same experimental model, performed by Balistreri et al., showed the similar properties of another synthetic cannabinoid labeled WIN55,212-2, a full CB1 agonist that prevents dermal fibrosis by inhibiting TGF-β as well as connective tissue growth factor and platelet-derived growth factor [178]. Mast cell downregulation, alongside a decrease in IL-4 levels, was also reported in an in vivo model for ACD induced with oxazolone on Balb/c and hairless mice that were treated with a topical synthetic CB1 agonist named α-oleoyl oleylamine serinol (α-OOS) [179].

Topically applied cannabidiol has also demonstrated anti-inflammatory and analgesic effects by inhibiting cyclooxygenase and lipoxygenase, properties that were observed by Formukong et al. in an experimental model of the tetradecanoylphorbol acetate-induced erythema of mouse skin performed decades ago, a study which represented a basis for more advanced research and unlocked the potential for the application of cannabinoids in acne, ACD, dermatomyositis, and other inflammatory skin disorders [180].

A suspected yet unconfirmed consequence of the anti-inflammatory roles of cannabinoids is the decrease of tumor viability, proliferation and growth in cutaneous melanoma. Armstrong et al. studied a combination of THC and CBD in a cutaneous melanoma model on mice bearing BRAF wild-type melanoma xenografts and found that the product caused a reduction of reactive oxygen species production and caspase activation, effects that may be triggered by the anti-inflammatory effects of CBD [181]. Skin tumorigenesis is an intricate process that depends on molecules common to the inflammatory signaling pathways, entailing complex interferences induced by substances such as matrix metalloproteinases, TNF-α and IL-1 that are implicated in inflammation as well as a wide array of malignancies, including skin cancer [182,183,184,185,186].

The decreased expression of TNF-α and NF-κB triggered by cannabinoids noted in in vitro studies was also demonstrated in an in vivo study on the CB receptors in the inflammatory milieu of skin cancer. Zheng et al. showed that CB receptors play a role in the resistance to ultraviolet (UV) B inflammation in an in vivo model of UV-induced skin carcinogenesis, and that CB receptor deficiency correlates with an increase in the expression of TNF-α, and the activation of NF-κB and mitogen-activated protein MAP kinases [187].

### 6.3. Human Clinical Trials

Developing on the favorable results cited in multiple in vivo studies, Ali et al. tested the safety and efficacy of an extract cream made of cannabis seeds in healthy patients and noted significant sebum diminution and erythema reduction while noting possible skin irritation as an adverse effect [188]. A Phase 2 study ran by Botanix Pharmaceuticals evaluating a proprietary synthetic CBD-derived cannabinoid labeled BTX 1503 on more than 350 patients with acne was recently completed, but the results are not yet available [189].

Cannabinoids were clinically tested in diffuse cutaneous systemic sclerosis, in a double-blind, randomized placebo-controlled study on 42 subjects performed by Spiera et al. [190]. The authors noted that while the side effects included fatigue and vertigo, the patients receiving ajulemic acid had a significant improvement in their clinical scores, with a reduction in the expression of key genes related to inflammation on skin biopsies. Subsequently, a Phase 3 trial was launched in 2018, with an estimated 350 patients receiving either ajulemic acid in 20 mg or 5 mg concentrations, or a placebo [191].

Ajulemic acid was also tested in another Phase 2 trial conducted by Chen et al. for the effectiveness in controlling the inflammation in patients with dermatomyositis [192]. The oral administration of ajulemic acid leads to a decrease of IL-31, IFN-β and -γ, and T-helper cell inflammation, demonstrating effectiveness in the treatment of dermatomyositis skin lesions. Werth et al. developed a Phase 3 study to test the performance of ajulemic acid in dermatomyositis [193].

## 7. Integrative Vision on the Effects of Cannabinoids in Gut-Lung-Skin Epithelial Inflammation

In summary, in a multitude of inflammatory models located in the epithelia of the gut-lung-skin barrier, cannabinoids show similar and consistent effects. In vitro studies show that all types of cannabinoids have potent anti-inflammatory effects, mostly expressed as the ability to prevent a rise in cytokines when inflammation is produced in the cellular experimental model (Table 1). Consistently, both natural and artificial cannabinoids were able to regulate the induced increase in the expression of TNF-α, IFN-γ, Ils 1β, 6, 8 and 17A, as well as MCP-1 and 2, and various other cofactors of inflammation. Vascular and cellular proliferative factors were also suppressed, adding to the potential benefits in regulating inflammation, possibly including the processes in the tumoral microenvironment. While some controversial results were documented, there is a large number of studies supporting the positive effects of cannabinoids in inflammations of the gut-lung-skin axis, and some significant results are presented in Table 1.

The investigation of the effects of cannabinoids on animal models of various inflammatory conditions of the gut-lung-skin axis has validated the anticipated results. Cannabinoids are able to reproduce the same anti-inflammatory effects that were observed in vitro in advanced experimental in vivo models, which lead to the identification of further beneficial effects. Cannabinoids modulate cell signaling and regulate the accumulation of inflammatory cells at the site of the inflammation, while also decreasing the activity of some chemokines and the expression of pro-inflammatory proteins. All of these positive direct and indirect anti-inflammatory effects have been attributed to a wide variety of cannabinoids, and relevant studies that support these findings are listed in Table 2.

The encouraging results emerging from in vitro and animal studies have prompted the translation to human trials. However, this has been made difficult, at least in part, by legislation and the need to obtain authorization from institutions regulating the use of cannabinoids that need to enforce national, trans-national and international regulations [194]. However, multiple trials have explored the potential of cannabinoids in inflammatory diseases pertaining to the gut-lung-skin barrier. The status of these trials and the preliminary or definitive results are listed in Table 3.

**Figure 1:**
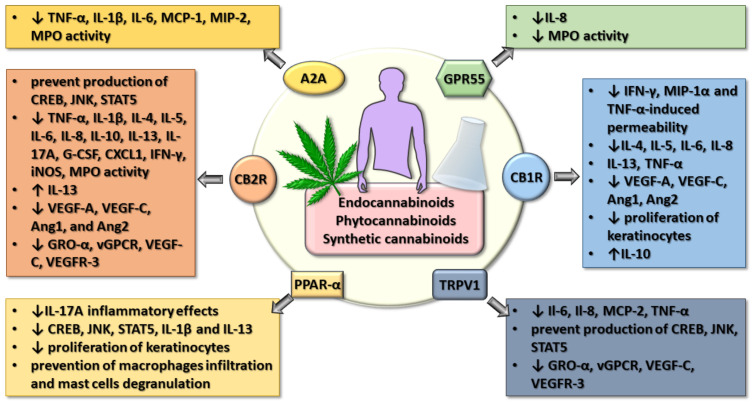
The summarization of the main effects of endocannabinoids, as well as natural and synthetic cannabinoids on several receptors promoting the decrease of inflammation in the gut-lung-skin barrier. (↓) = decrease in expression and/or concentration; (↑) = increase in expression and/or concentration.

Unfortunately, not all of the cannabinoids that have proven effective in in vitro conditions or on animal models have provided significant results in humans, and some even presented significant adverse effects. Nevertheless, perfecting some new artificial cannabinoids with optimal receptor affinities or associating other compounds that modulate the effects via antagonism or post-receptor regulation may lead to improved results. The higher patient acceptance of natural compounds or their derivatives may prove essential in obtaining better compliance and superior results when associating cannabinoids in inflammatory conditions, especially in a chronic setting which implies sustained treatment [195]. Further randomized placebo-controlled clinical trials are needed to support the already overwhelming evidence regarding the beneficial results of cannabinoids in inflammations of the gut-lung-skin epithelium. In Figure 1, we summarized the effects of natural or synthetic cannabinoids on several receptors implicated in decreasing the inflammation in the gut-lung-skin barrier.

## 8. Conclusions

The epithelia of the gastrointestinal tract, the pulmonary system and the cutaneous membrane share common features in regard to physiology, molecular signaling, and cell activity in pathological conditions. Furthermore, recent data suggest that the gut-lung-skin barrier exhibits cross-talk involving various cells and cytokines, and responds in a similar way to epithelial disruption and local inflammation. This unitary behavior is also evidenced in the response to cannabinoids, a large class of compounds with well-known anti-inflammatory properties. A large number of in vitro and in vivo animal studies have shown promising results in either reducing or preventing inflammation in experimental models involving the elements of the gut-lung-skin barrier. Human studies have shown mixed results, with some cannabinoids either being ineffective or not tolerable in the treatment of specific inflammatory conditions. The reasons for treatment failure or intolerance may be revealed by translating the observations regarding the effects of cannabinoids on inflammation between studies involving specific elements of the gut-lung-skin barrier. The success that some of the clinical trials have achieved is encouraging and warrants further studies that will undoubtedly clarify the definitive role of cannabinoids in epithelial inflammation.

## Figures and Tables

**Table 1:** The roles of cannabinoids in inflammatory conditions of the gut-lung-skin barrier demonstrated in in vitro studies.

Cannabinoid	Impacted Molecules	Receptors/Pathway	Experimental Model	Study
AEA and CBD	IL-17A inflammatory effects (↓)	Mostly CB2, possibly PPAR- γ-mediated effects	Confluent Caco-2 cell monolayers	Harvey et al. [57]
THC and CBD	IFN-γ and TNF-α-induced permeability (↓)	CB1-mediated effects	Confluent Caco-2 cell monolayers	Alhamoruni et al. [58]
AEA and 2-AG	IFN-γ and TNF-α-induced permeability (↑)	CB1-mediated effects	Confluent Caco-2 cell monolayers	Alhamoruni et al. [58]
AEA	IL-6, IL-8 (↓)	CB1-mediated effects, possibly TRPV1 modulation	Caco-2 cell monolayers	Karwad et al. [60]
CBD and PEA	prevents production of CREB, JNK, STAT5	CB2, TRPV1 (CBD) PPAR-α (PEA)	Caco-2 cells	Couch et al. [61]
CBD and THCA	IL-8 (↓)	partially GPR55-mediated (THCA)	HCT116, HT29, and Caco-2 colon cells	Nallathambi et al. [62]
BPC	TNF-α, IL-1β, IL-6, IL-17A (↓) IL-13 (↑)	CB2-mediated STAT-3 downregulation	Human gingival fibroblasts and mucosa epithelial cells	Picciolo et al. [65]
CBD	IL-6, G-CSF, CXCL1 (↓) IL-8, GM-CSF, CXCL2 (↑)	Unspecified, possibly CB2-mediated	Normal bronchial cells treated with TNF-α	Muthumalage et al. [111]
CBD	MCP-1 (↓)	NF-κB inhibition	BEAS-2B, U937, and HFL-1 cells	Muthumalage et al. [111]
ACEA and JWH-133	VEGF-A, VEGF-C, Ang1, and Ang2 (↓)	CB1 (ACEA) CB2 (JWH-133)	Human lung macrophages	Staiano et al. [113]
AJA	TNF-α, IFN-α and β (↓)	Unspecified, possibly CB2-mediated	Peripheral blood mononuclear cells from dermatomyositis patients	Robinson et al. [173]
PEA	MCP-2 (↓)	“Entourage” effect on TRPV1 ligands (AEA and OEA)	HaCaT keratinocytes treated with polyinosinic:polycytidylic acid	Petrosino et al. [168]
CBD	MCP-2, IL-6, IL-8, TNF-α (↓)	CB2-mediated as well as through TRPV1 activation/desensitization	HaCaT keratinocytes treated with polyinosinic:polycytidylic acid	Petrosino et al. [169]
THC, CBN, CBD, and CBG	Keratinocytes (inhibits proliferation)	Predominantly mediated by PPAR-γ	HPV-16 E6/E7 transformed human skin keratinocytes	Wilkinson et al. [171]
ACEA	keratinocytes (inhibits proliferation)	CB1-mediated signaling	Isolated human skin samples of psoriasis lesions	Ramot et al. [172]
CBD	GRO-α, vGPCR, VEGF-C, VEGFR-3 (↓)	Unspecified, possibly mediated by multiple receptors (CB1, CB2, TRPVs, and/or GPRs)	Kaposi’s sarcoma-associated herpesvirus-infected primary human dermal endothelial cells	Maor et al. [175]
CBD	TNF-α, IL-1β, IL-6 (↓)	A2a adenosine receptor-cAMP-TRIB3-NF-κB pathway	SZ95 human sebocytes cultures	Oláh et al. [174]

(↓) = decrease in expression and/or concentration; (↑) = increase in expression and/or concentration; BPC = β-Caryophyllene; OEA = oleoylethanolamide; TRPV = transient receptor potential channel; GPR = G-coupled protein receptor.

**Table 2:** The roles of cannabinoids in inflammatory conditions of the gut-lung-skin barrier evidenced in in vivo studies.

Cannabinoid	Impacted Molecules	Receptors/Pathway	Experimental Model	Study
CBG	IL-1β, IL-10, IFN-γ, iNOS expression, MPO activity (↓)	CB2 and possibly TRPV4-mediated	Murine colitis induced by DNBS	Borrelli et al. [70]
CBD	IL-1β, IL-10, iNOS expression (↓)	Unspecified, possibly CB2-mediated	Murine colitis induced by DNBS	Borrelli et al. [71]
CBD and CBG combined with fish oil	IL-1β, MPO activity (↓)	Possibly by regulating endocannabinoids and their derivates	Murine colitis induced by DNBS	Pagano et al. [72]
CBD combined with fish oil	IL-1β, IL-6, MPO activity (↓) IL-10 (↑)	*Unspecified*	DSS model of murine colitis	Silvestri et al. [73]
Abn-CBD	MPO activity (↓)	Non-CB1/2, possibly GPR18 and GPR55	TNBS-induced colitis in CD1 mice	Krohn et al. [80]
HU210	IL-1β, IL-6, IL-17, TNF-α, MPO activity (↓)	TLR4/MAPK signaling pathway	DSS model of murine colitis	Lin et al. [79]
HU210	IL-6, chemokine KC (↓)	CB1/2 receptor agonism	Gastric mucosa inflammation secondary to acute pancreatitis in rats	Cao et al. [78]
CBD	IL-6, TNF-α, MCP-1, MIP-2, MPO activity (↓)	Non-CB1/2, possibly through the adenosine A2A receptor	Lipopolysaccharide-induced acute lung injury in mice	Ribeiro et al. [117,118]
WIN 55,212-2, PEA and THC	TNF-α (↓)	Partially CB2-mediated	Lipopolysaccharide-induced acute lung injury in mice	Beryshev et al. [119]
CBD	IL-5, IL-23, G-CSF, TNF-α (↑)	Increased activation of NFAT and Ca^2+^ signaling	Lipopolysaccharide-induced acute lung injury in mice	Karmaus et al. [120]
CBD	IL-1 and total protein content (↓)	5-HT1A receptor	Lung inflammation induced by brain ischemia in newborn piglets	Arruza et al. [121]
WIN55,212-2	MMP-9 (↓)	ERK1/2 signaling pathway	Lung inflammation in mice exposed to cigarette smoke	Tauber et al. [122]
CBD	IL-4, IL-5, IL-13, IL-6, and TNF-α (↓)	CB1/2-mediated	Ovalbumin-induced asthma in mice	Vuolo et al. [123,124]
JWH-133	CD11b surface expression/adhesion, ROS production (↑)	CB2-mediated	Ovalbumin-induced asthma in mice	Frei et al. [125]
JWH-133 combined with JZL184	IFN-γ, MIP-1α (↓) IL-10 (↑)	CB1 (JZL184) CB2 (JWH-133)	Lung inflammation in RSV infection in mice	Tahamtan et al. [127,128]
THC	IFN-γ (↓)	PI3K/Akt pathway signaling inhibition	Murine model of lung injury caused by SEB	Rao et al. [131]
JWH-133	IL-6, TNF-α, MPO activity (↓) SOD activity (↑)	CB2-mediated activation of PI3K/Akt pathway signaling	Lung ischemia-reperfusion injury model in mice	Zeng et al. [132]
JWH-133	IL-1β, IL-6, TNF-α, caspase-3 (↓) IL-10 (↑)	NF-κB signaling inhibition	Mice with CLP-induced sepsis	Çakır et al. [133]
GP1a	IL-6, chemokine KC, MIP-2 (↓)	CB2-mediated	Mice with CLP-induced sepsis	Tschöp et al. [134]
M. suaveolens	IL-1β, IL-6, TNF-α, VEGF (↓) IL-4, IL-10 (↑)	NF-κB signaling inhibition	Mice with CLP-induced sepsis	Liu et al. [135]
JWH-133	MPO activity (↓)	CB2-mediated	Murine model of NPE after subarachnoid hemorrhage	Fujii et al. [136]
BPC and GP1a	IL-12, chemokine KC, leukotriene B_4_, NO (↓)	CB2-mediated	Mycobacterium bovis-induced pulmonary inflammation	Andrade-Silva et al. [138]
Possible unspecified agonist *	TNF-α, NF-κB and MAP kinases * (↓)	CB1 and CB2-mediated NF-κB and MAP/ERK signaling	Murine UV-induced skin carcinogenesis	Zheng et al. [187]
VCE-004.8	IL-1β and IL-13. Prevention of macrophages infiltration and mast cells degranulation. Indirectly decreased TGF-β	PPAR-γ and CB2-mediated SMAD-signaling transcriptional activity modulation	Mice with bleomycin-induced scleroderma	del Río et al. [177]
WIN55,212-2	TGF-β, CTGF, and PDGF (↓)	Non-CB1, non-CB2 mediated downregulation of PDGF/TGFβ signaling pathways	Mice with bleomycin-induced scleroderma	Balistreri et al. [178]
α-OOS	Mast cells degranulation and IL-4 (↓)	“Entourage” effect on CB1 with possible PPAR-γ and GPR55 involvement	Oxazolone induced atopic dermatitis in Balb/c and hairless mice	Kim et al. [179]
CBD	Arachidonate (↑) and prostaglandins (↓)	Increased PLA_2_ activity and inhibition of cyclooxygenase and lipoxygenase	Tetradecanoylphorbol acetate-induced erythema of mouse skin	Formukong et al. [180]
CBD	ROS production and caspase activation (↓)	Undetermined anti-inflammatory pathway *. TRIB3-Akt/mTOR signaling pathway	Mice bearing BRAF wild-type melanoma xenografts	Armstrong et al. [181]

* Hypothesized, yet to be demonstrated; (↓) = decrease in expression and/or concentration; (↑) = increase in expression and/or concentration; CLP = cecal ligation and puncture; DNBS = 2,4-dinitrobenzene sulphonic acid; DSS = dextran sulphate sodium; PLA_2_ = phospholipase A_2_; MDA = malondialdehyde; MPO = myeloperoxidase; mTOR = mammalian target of rapamycin; NFAT = nuclear factor of activated T cells; NPE = neurogenic pulmonary edema; TNBS = trinitrobenzene sulfonic acid; RSV = respiratory syncytial virus; SEB = taphylococcal enterotoxin B; SOD = superoxide dismutase.

**Table 3:** Human trials evaluating the safety and efficacy of cannabinoids in inflammatory conditions of the gut-lung-skin barrier.

Cannabinoid	Effects	Inflammatory Condition	Clinical Trial Stage	Study
PEA and CBD	Decreased intestinal permeability	Aspirin-induced intestinal inflammation	Phase 1	Couch et al. [87]
THC	Decreased CDAI and CRP Increased quality of life	Crohn’s disease	Phase 1	Naftali et al. [88]
CBD	No beneficial effects	Crohn’s disease	Phase 2	Naftali et al. [89]
CBD	Minor improvements in rectal bleeding and endoscopic scores. Increased quality of life	Ulcerative colitis	Phase 2	Irving et al. [91]
THC	Decreased DAI and endoscopic score	Ulcerative colitis	Phase 1	Naftali et al. [92]
Lenabasum	Decreased IgG, IL-8, exacerbations, and lymphocyte infiltration	Cystic fibrosis	Phase 2	Chmiel et al. [143]
Cannabis (high CBD/low THC)	*Results pending*	Pain and Inflammation in Lung Cancer	Phase 1	Martinez et al. [144]
Cannabis (high CBD/low THC)	No clinical positive or negative effects	Chronic obstructive pulmonary disease	Phase 2	Abdallah et al. [146]
Nabilone	No significant bronchodilation	Asthma	Phase 1	Gong et al. [147]
THC	Antagonistic effects on bronchodilation. Irritating effect on airways	Asthma	Phase 1	Tashkin et al. [148]
THC	Mild bronchodilation effect. Significant psychoactive effects	Asthma	Phase 1	Abboud et al. [149]
Cannabis seeds mixture	Decreased erythema	Acne	Phase 1	Ali et al. [188]
BTX 1503	*Results pending*	Acne	Phase 2	Botanix Pharmaceuticals [189]
AJA	Decreased expression of key genes related to inflammation	Cutaneous systemic sclerosis	Phase 2	Spiera et al. [190]
AJA	Decreased IL-31, IFN-β and γ, and T-helper cell inflammation	Dermatomyositis	Phase 2	Chen et al. [192]
AJA	*Initiated study*	Cutaneous systemic sclerosis	Phase 3	Burstein et al. [191]
AJA	*Developed study protocol*	Dermatomyositis	Phase 3	Werth et al. [193]

CDAI = Crohn’s disease activity index; CRP = C-reactive protein; DAI = disease activity index; IgG = immunoglobulin G.

## Data Availability

Not applicable.
